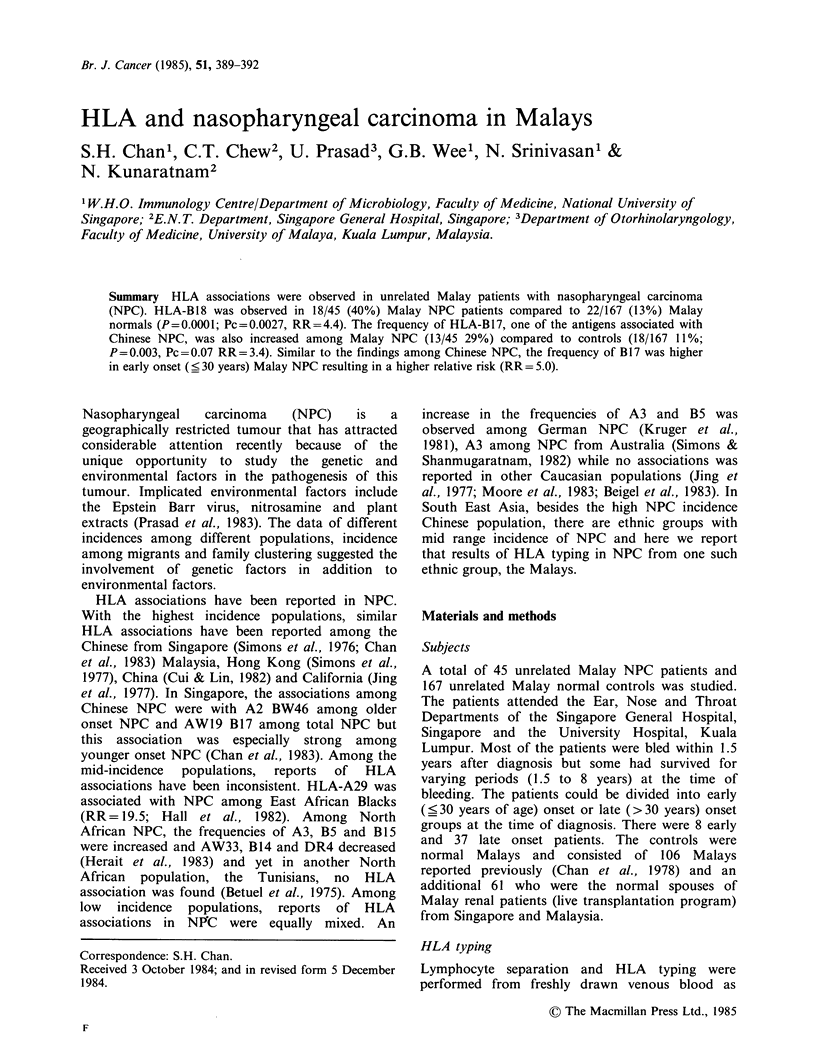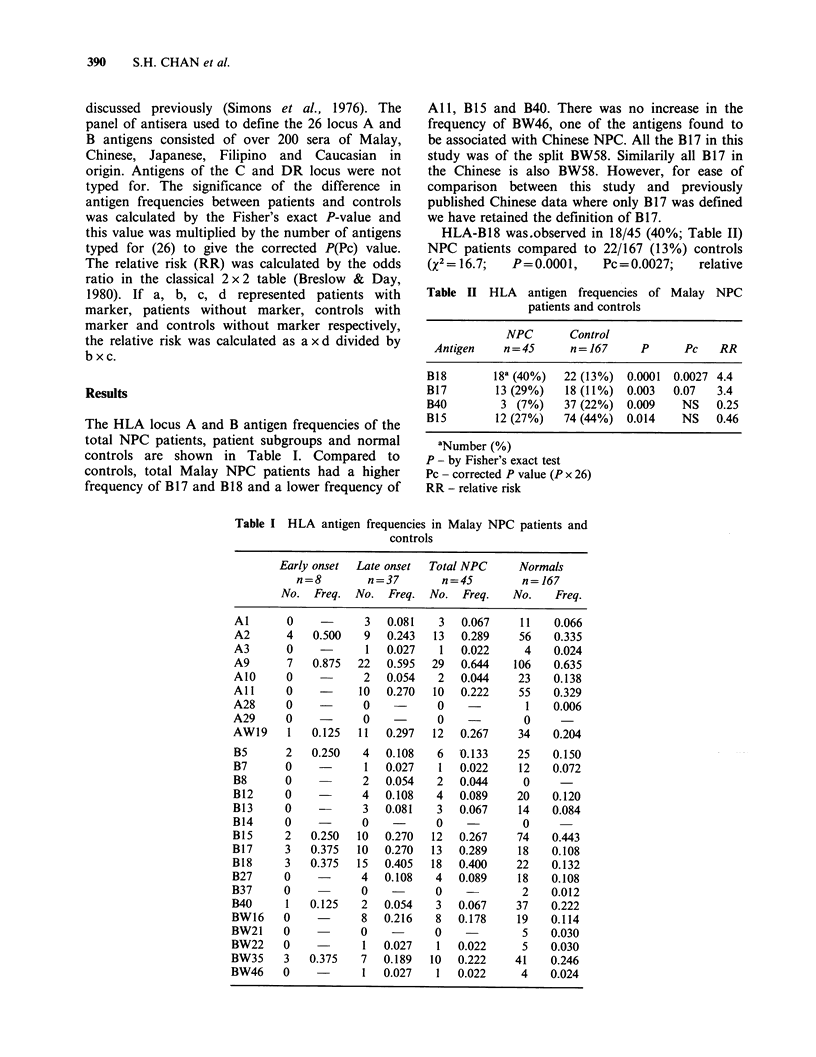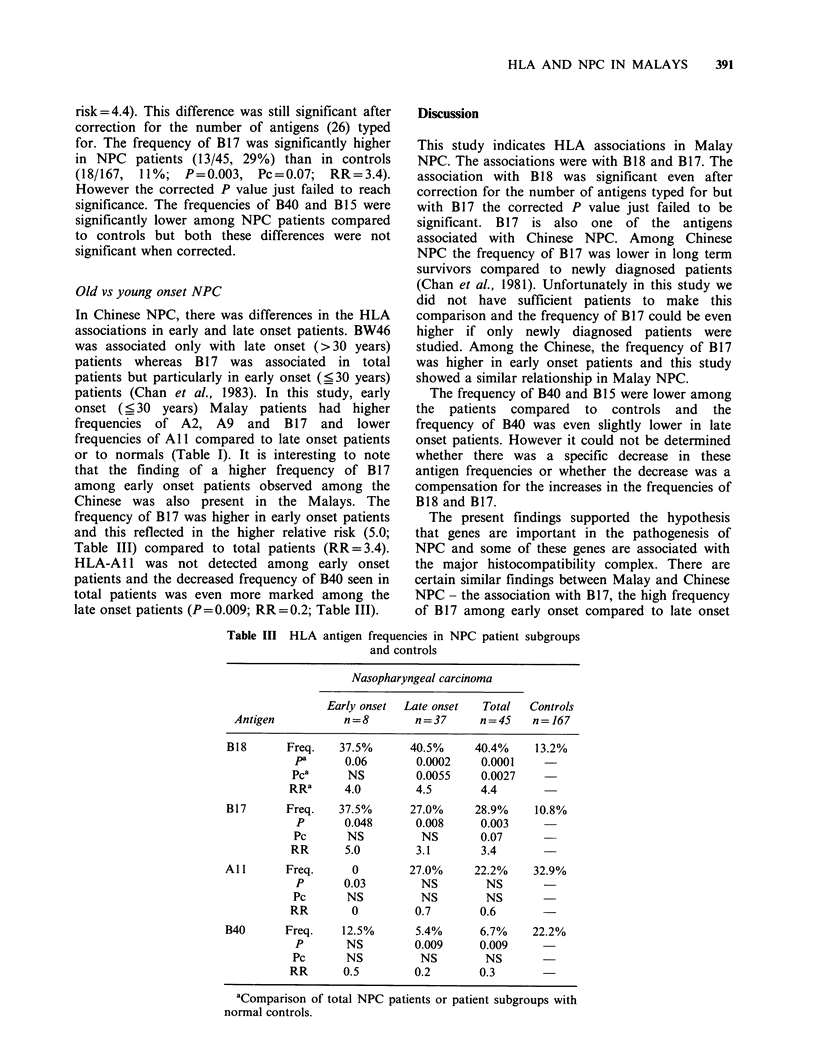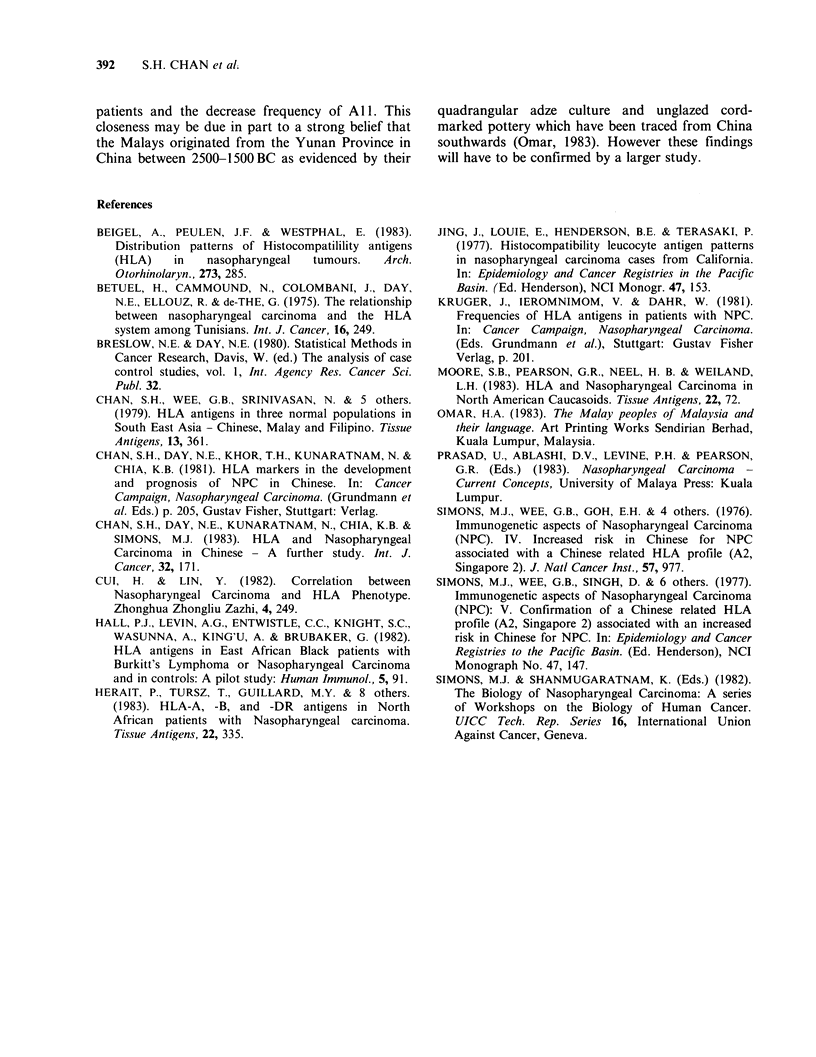# HLA and nasopharyngeal carcinoma in Malays.

**DOI:** 10.1038/bjc.1985.52

**Published:** 1985-03

**Authors:** S. H. Chan, C. T. Chew, U. Prasad, G. B. Wee, N. Srinivasan, N. Kunaratnam

## Abstract

HLA associations were observed in unrelated Malay patients with nasopharyngeal carcinoma (NPC). HLA-B18 was observed in 18/45 (40%) Malay NPC patients compared to 22/167 (13%) Malay normals (P = 0.0001; Pc = 0.0027, RR = 4.4). The frequency of HLA-B17, one of the antigens associated with Chinese NPC, was also increased among Malay NPC (13/45 29%) compared to controls (18/167 11%; P = 0.003, Pc = 0.07 RR = 3.4). Similar to the findings among Chinese NPC, the frequency of B17 was higher in early onset (less than or equal to 30 years) Malay NPC resulting in a higher relative risk (RR = 5.0).


*Br. J. Cancer* (1985), **51**, 389–392

### HLA and nasopharyngeal carcinoma in Malays

S.H. Chan[1], C.T. Chew[2], U. Prasad[3], G.B. Wee[1], N. Srinivasan[1] & N. Kunaratnam[2]

[1]*W.H.O. Immunology Centre/Department of Microbiology, Faculty of Medicine, National University of Singapore;* [2]*E.N.T. Department, Singapore General Hospital, Singapore;* [3]*Department of Otorhinolaryngology, Faculty of Medicine, University of Malaya, Kuala Lumpur, Malaysia.*

**Summary** HLA associations were observed in unrelated Malay patients with nasopharyngeal carcinoma (NPC). HLA-B18 was observed in 18/45 (40%) Malay NPC patients compared to 22/167 (13%) Malay normals ($P=0.0001$; Pc=0.0027, RR=4.4). The frequency of HLA-B17, one of the antigens associated with Chinese NPC, was also increased among Malay NPC (13/45 29%) compared to controls (18/167 11%; $P=0.003$, Pc=0.07 RR=3.4). Similar to the findings among Chinese NPC, the frequency of B17 was higher in early onset ($\leq 30$ years) Malay NPC resulting in a higher relative risk (RR=5.0).

Nasopharyngeal carcinoma (NPC) is a geographically restricted tumour that has attracted considerable attention recently because of the unique opportunity to study the genetic and environmental factors in the pathogenesis of this tumour. Implicated environmental factors include the Epstein Barr virus, nitrosamine and plant extracts (Prasad *et al.*, 1983). The data of different incidences among different populations, incidence among migrants and family clustering suggested the involvement of genetic factors in addition to environmental factors.

HLA associations have been reported in NPC. With the highest incidence populations, similar HLA associations have been reported among the Chinese from Singapore (Simons *et al.*, 1976; Chan *et al.*, 1983) Malaysia, Hong Kong (Simons *et al.*, 1977), China (Cui & Lin, 1982) and California (Jing *et al.*, 1977). In Singapore, the associations among Chinese NPC were with A2 BW46 among older onset NPC and AW19 B17 among total NPC but this association was especially strong among younger onset NPC (Chan *et al.*, 1983). Among the mid-incidence populations, reports of HLA associations have been inconsistent. HLA-A29 was associated with NPC among East African Blacks (RR=19.5; Hall *et al.*, 1982). Among North African NPC, the frequencies of A3, B5 and B15 were increased and AW33, B14 and DR4 decreased (Herait *et al.*, 1983) and yet in another North African population, the Tunisians, no HLA association was found (Betuel *et al.*, 1975). Among low incidence populations, reports of HLA associations in NPC were equally mixed. An increase in the frequencies of A3 and B5 was observed among German NPC (Kruger *et al.*, 1981), A3 among NPC from Australia (Simons & Shanmugaratnam, 1982) while no associations was reported in other Caucasian populations (Jing *et al.*, 1977; Moore *et al.*, 1983; Beigel *et al.*, 1983). In South East Asia, besides the high NPC incidence Chinese population, there are ethnic groups with mid range incidence of NPC and here we report that results of HLA typing in NPC from one such ethnic group, the Malays.

### Materials and methods

### *Subjects*

A total of 45 unrelated Malay NPC patients and 167 unrelated Malay normal controls was studied. The patients attended the Ear, Nose and Throat Departments of the Singapore General Hospital, Singapore and the University Hospital, Kuala Lumpur. Most of the patients were bled within 1.5 years after diagnosis but some had survived for varying periods (1.5 to 8 years) at the time of bleeding. The patients could be divided into early ($\leq 30$ years of age) onset or late ($>30$ years) onset groups at the time of diagnosis. There were 8 early and 37 late onset patients. The controls were normal Malays and consisted of 106 Malays reported previously (Chan *et al.*, 1978) and an additional 61 who were the normal spouses of Malay renal patients (live transplantation program) from Singapore and Malaysia.

### *HLA typing*

Lymphocyte separation and HLA typing were performed from freshly drawn venous blood as

Correspondence: S.H. Chan.
Received 3 October 1984; and in revised form 5 December 1984.

© The Macmillan Press Ltd., 1985

discussed previously (Simons et al., 1976). The panel of antisera used to define the 26 locus A and B antigens consisted of over 200 sera of Malay, Chinese, Japanese, Filipino and Caucasian in origin. Antigens of the C and DR locus were not typed for. The significance of the difference in antigen frequencies between patients and controls was calculated by the Fisher's exact $P$-value and this value was multiplied by the number of antigens typed for (26) to give the corrected $P$(Pc) value. The relative risk (RR) was calculated by the odds ratio in the classical 2 × 2 table (Breslow & Day, 1980). If a, b, c, d represented patients with marker, patients without marker, controls with marker and controls without marker respectively, the relative risk was calculated as a × d divided by b × c.

### Results

The HLA locus A and B antigen frequencies of the total NPC patients, patient subgroups and normal controls are shown in Table I. Compared to controls, total Malay NPC patients had a higher frequency of B17 and B18 and a lower frequency of A11, B15 and B40. There was no increase in the frequency of BW46, one of the antigens found to be associated with Chinese NPC. All the B17 in this study was of the split BW58. Similarily all B17 in the Chinese is also BW58. However, for ease of comparison between this study and previously published Chinese data where only B17 was defined we have retained the definition of B17.

HLA-B18 was.observed in 18/45 (40%; Table II) NPC patients compared to 22/167 (13%) controls ($\chi^2 = 16.7$; $P = 0.0001$, Pc = 0.0027; relative

**Table II** HLA antigen frequencies of Malay NPC patients and controls

| *Antigen* | *NPC n=45* | *Control n=167* | *P* | *Pc* | *RR* |
|---|---|---|---|---|---|
| B18 | 18[a] (40%) | 22 (13%) | 0.0001 | 0.0027 | 4.4 |
| B17 | 13 (29%) | 18 (11%) | 0.003 | 0.07 | 3.4 |
| B40 | 3 (7%) | 37 (22%) | 0.009 | NS | 0.25 |
| B15 | 12 (27%) | 74 (44%) | 0.014 | NS | 0.46 |

[a]Number (%)
$P$ – by Fisher's exact test
Pc – corrected $P$ value ($P \times 26$)
RR – relative risk

**Table I** HLA antigen frequencies in Malay NPC patients and controls

| | *Early onset n=8* | | *Late onset n=37* | | *Total NPC n=45* | | *Normals n=167* | |
|---|---|---|---|---|---|---|---|---|
| | *No.* | *Freq.* | *No.* | *Freq.* | *No.* | *Freq.* | *No.* | *Freq.* |
| A1 | 0 | — | 3 | 0.081 | 3 | 0.067 | 11 | 0.066 |
| A2 | 4 | 0.500 | 9 | 0.243 | 13 | 0.289 | 56 | 0.335 |
| A3 | 0 | — | 1 | 0.027 | 1 | 0.022 | 4 | 0.024 |
| A9 | 7 | 0.875 | 22 | 0.595 | 29 | 0.644 | 106 | 0.635 |
| A10 | 0 | — | 2 | 0.054 | 2 | 0.044 | 23 | 0.138 |
| A11 | 0 | — | 10 | 0.270 | 10 | 0.222 | 55 | 0.329 |
| A28 | 0 | — | 0 | — | 0 | — | 1 | 0.006 |
| A29 | 0 | — | 0 | — | 0 | — | 0 | — |
| AW19 | 1 | 0.125 | 11 | 0.297 | 12 | 0.267 | 34 | 0.204 |
| B5 | 2 | 0.250 | 4 | 0.108 | 6 | 0.133 | 25 | 0.150 |
| B7 | 0 | — | 1 | 0.027 | 1 | 0.022 | 12 | 0.072 |
| B8 | 0 | — | 2 | 0.054 | 2 | 0.044 | 0 | — |
| B12 | 0 | — | 4 | 0.108 | 4 | 0.089 | 20 | 0.120 |
| B13 | 0 | — | 3 | 0.081 | 3 | 0.067 | 14 | 0.084 |
| B14 | 0 | — | 0 | — | 0 | — | 0 | — |
| B15 | 2 | 0.250 | 10 | 0.270 | 12 | 0.267 | 74 | 0.443 |
| B17 | 3 | 0.375 | 10 | 0.270 | 13 | 0.289 | 18 | 0.108 |
| B18 | 3 | 0.375 | 15 | 0.405 | 18 | 0.400 | 22 | 0.132 |
| B27 | 0 | — | 4 | 0.108 | 4 | 0.089 | 18 | 0.108 |
| B37 | 0 | — | 0 | — | 0 | — | 2 | 0.012 |
| B40 | 1 | 0.125 | 2 | 0.054 | 3 | 0.067 | 37 | 0.222 |
| BW16 | 0 | — | 8 | 0.216 | 8 | 0.178 | 19 | 0.114 |
| BW21 | 0 | — | 0 | — | 0 | — | 5 | 0.030 |
| BW22 | 0 | — | 1 | 0.027 | 1 | 0.022 | 5 | 0.030 |
| BW35 | 3 | 0.375 | 7 | 0.189 | 10 | 0.222 | 41 | 0.246 |
| BW46 | 0 | — | 1 | 0.027 | 1 | 0.022 | 4 | 0.024 |

risk = 4.4). This difference was still significant after correction for the number of antigens (26) typed for. The frequency of B17 was significantly higher in NPC patients (13/45, 29%) than in controls (18/167, 11%; $P = 0.003$, Pc = 0.07; RR = 3.4). However the corrected $P$ value just failed to reach significance. The frequencies of B40 and B15 were significantly lower among NPC patients compared to controls but both these differences were not significant when corrected.

*Old vs young onset NPC*

In Chinese NPC, there was differences in the HLA associations in early and late onset patients. BW46 was associated only with late onset (>30 years) patients whereas B17 was associated in total patients but particularly in early onset (≦30 years) patients (Chan *et al.*, 1983). In this study, early onset (≦30 years) Malay patients had higher frequencies of A2, A9 and B17 and lower frequencies of A11 compared to late onset patients or to normals (Table I). It is interesting to note that the finding of a higher frequency of B17 among early onset patients observed among the Chinese was also present in the Malays. The frequency of B17 was higher in early onset patients and this reflected in the higher relative risk (5.0; Table III) compared to total patients (RR = 3.4). HLA-A11 was not detected among early onset patients and the decreased frequency of B40 seen in total patients was even more marked among the late onset patients ($P = 0.009$; RR = 0.2; Table III).

### Discussion

This study indicates HLA associations in Malay NPC. The associations were with B18 and B17. The association with B18 was significant even after correction for the number of antigens typed for but with B17 the corrected $P$ value just failed to be significant. B17 is also one of the antigens associated with Chinese NPC. Among Chinese NPC the frequency of B17 was lower in long term survivors compared to newly diagnosed patients (Chan *et al.*, 1981). Unfortunately in this study we did not have sufficient patients to make this comparison and the frequency of B17 could be even higher if only newly diagnosed patients were studied. Among the Chinese, the frequency of B17 was higher in early onset patients and this study showed a similar relationship in Malay NPC.

The frequency of B40 and B15 were lower among the patients compared to controls and the frequency of B40 was even slightly lower in late onset patients. However it could not be determined whether there was a specific decrease in these antigen frequencies or whether the decrease was a compensation for the increases in the frequencies of B18 and B17.

The present findings supported the hypothesis that genes are important in the pathogenesis of NPC and some of these genes are associated with the major histocompatibility complex. There are certain similar findings between Malay and Chinese NPC – the association with B17, the high frequency of B17 among early onset compared to late onset

**Table III** HLA antigen frequencies in NPC patient subgroups and controls

| | | *Nasopharyngeal carcinoma* | | | |
|---|---|---|---|---|---|
| *Antigen* | | *Early onset* *n = 8* | *Late onset* *n = 37* | *Total* *n = 45* | *Controls* *n = 167* |
| B18 | Freq. | 37.5% | 40.5% | 40.4% | 13.2% |
| | $P^a$ | 0.06 | 0.0002 | 0.0001 | — |
| | Pc[a] | NS | 0.0055 | 0.0027 | — |
| | RR[a] | 4.0 | 4.5 | 4.4 | — |
| B17 | Freq. | 37.5% | 27.0% | 28.9% | 10.8% |
| | $P$ | 0.048 | 0.008 | 0.003 | — |
| | Pc | NS | NS | 0.07 | — |
| | RR | 5.0 | 3.1 | 3.4 | — |
| A11 | Freq. | 0 | 27.0% | 22.2% | 32.9% |
| | $P$ | 0.03 | NS | NS | — |
| | Pc | NS | NS | NS | — |
| | RR | 0 | 0.7 | 0.6 | — |
| B40 | Freq. | 12.5% | 5.4% | 6.7% | 22.2% |
| | $P$ | NS | 0.009 | 0.009 | — |
| | Pc | NS | NS | NS | — |
| | RR | 0.5 | 0.2 | 0.3 | — |

[a]Comparison of total NPC patients or patient subgroups with normal controls.

patients and the decrease frequency of A11. This closeness may be due in part to a strong belief that the Malays originated from the Yunan Province in China between 2500–1500 BC as evidenced by their quadrangular adze culture and unglazed cord-marked pottery which have been traced from China southwards (Omar, 1983). However these findings will have to be confirmed by a larger study.

### References

BEIGEL, A., PEULEN, J.F. & WESTPHAL, E. (1983). Distribution patterns of Histocompatilility antigens (HLA) in nasopharyngeal tumours. *Arch. Otorhinolaryn.*, **273,** 285.

BETUEL, H., CAMMOUND, N., COLOMBANI, J., DAY, N.E., ELLOUZ, R. & de-THE, G. (1975). The relationship between nasopharyngeal carcinoma and the HLA system among Tunisians. *Int. J. Cancer,* **16,** 249.

BRESLOW, N.E. & DAY, N.E. (1980). Statistical Methods in Cancer Research, Davis, W. (ed.) The analysis of case control studies, vol. 1, *Int. Agency Res. Cancer Sci. Publ.* **32.**

CHAN, S.H., WEE, G.B., SRINIVASAN, N. & 5 others. (1979). HLA antigens in three normal populations in South East Asia – Chinese, Malay and Filipino. *Tissue Antigens,* **13,** 361.

CHAN, S.H., DAY, N.E., KHOR, T.H., KUNARATNAM, N. & CHIA, K.B. (1981). HLA markers in the development and prognosis of NPC in Chinese. In: *Cancer Campaign, Nasopharyngeal Carcinoma.* (Grundmann *et al.* Eds.) p. 205, Gustav Fisher, Stuttgart: Verlag.

CHAN, S.H., DAY, N.E., KUNARATNAM, N., CHIA, K.B. & SIMONS, M.J. (1983). HLA and Nasopharyngeal Carcinoma in Chinese – A further study. *Int. J. Cancer,* **32,** 171.

CUI, H. & LIN, Y. (1982). Correlation between Nasopharyngeal Carcinoma and HLA Phenotype. Zhonghua Zhongliu Zazhi, **4,** 249.

HALL, P.J., LEVIN, A.G., ENTWISTLE, C.C., KNIGHT, S.C., WASUNNA, A., KING'U, A. & BRUBAKER, G. (1982). HLA antigens in East African Black patients with Burkitt's Lymphoma or Nasopharyngeal Carcinoma and in controls: A pilot study: *Human Immunol.,* **5,** 91.

HERAIT, P., TURSZ, T., GUILLARD, M.Y. & 8 others. (1983). HLA-A, -B, and -DR antigens in North African patients with Nasopharyngeal carcinoma. *Tissue Antigens,* **22,** 335.

JING, J., LOUIE, E., HENDERSON, B.E. & TERASAKI, P. (1977). Histocompatibility leucocyte antigen patterns in nasopharyngeal carcinoma cases from California. In: *Epidemiology and Cancer Registries in the Pacific Basin.* (Ed. Henderson), NCI Monogr. **47,** 153.

KRUGER, J., IEROMNIMOM, V. & DAHR, W. (1981). Frequencies of HLA antigens in patients with NPC. In: *Cancer Campaign, Nasopharyngeal Carcinoma.* (Eds. Grundmann *et al.*), Stuttgart: Gustav Fisher Verlag, p. 201.

MOORE, S.B., PEARSON, G.R., NEEL, H. B. & WEILAND, L.H. (1983). HLA and Nasopharyngeal Carcinoma in North American Caucasoids. *Tissue Antigens,* **22,** 72.

OMAR, H.A. (1983). *The Malay peoples of Malaysia and their language.* Art Printing Works Sendirian Berhad, Kuala Lumpur, Malaysia.

PRASAD, U., ABLASHI, D.V., LEVINE, P.H. & PEARSON, G.R. (Eds.) (1983). *Nasopharyngeal Carcinoma – Current Concepts,* University of Malaya Press: Kuala Lumpur.

SIMONS, M.J., WEE, G.B., GOH, E.H. & 4 others. (1976). Immunogenetic aspects of Nasopharyngeal Carcinoma (NPC). IV. Increased risk in Chinese for NPC associated with a Chinese related HLA profile (A2, Singapore 2). *J. Natl Cancer Inst.,* **57,** 977.

SIMONS, M.J., WEE, G.B., SINGH, D. & 6 others. (1977). Immunogenetic aspects of Nasopharyngeal Carcinoma (NPC): V. Confirmation of a Chinese related HLA profile (A2, Singapore 2) associated with an increased risk in Chinese for NPC. In: *Epidemiology and Cancer Registries to the Pacific Basin.* (Ed. Henderson), NCI Monograph No. 47, 147.

SIMONS, M.J. & SHANMUGARATNAM, K. (Eds.) (1982). The Biology of Nasopharyngeal Carcinoma: A series of Workshops on the Biology of Human Cancer. *UICC Tech. Rep. Series* **16,** International Union Against Cancer, Geneva.